# Epilepsy-related stigma in Nigeria: A systematic review of manifestations, impacts, and socio-cultural drivers

**DOI:** 10.4102/ajod.v15i0.1875

**Published:** 2026-01-23

**Authors:** Boluwatife O. Alege, Chisom P. Agbo, Adeolu Anthony Olagunju

**Affiliations:** 1 Department of Public Health, Sport and Well-being, Faculty of Health, Medicine and Society, University of Chester, Chester, United Kingdom

**Keywords:** epilepsy, people living with epilepsy, stigma, discrimination, social exclusion, cultural beliefs, Nigeria

## Abstract

**Background:**

Stigma poses significant challenges to the overall quality of life of people living with epilepsy (PLWE) in Nigeria; yet, there remains a limited understanding of the stigmatisation of PLWE.

**Objectives:**

Guided by critical theory, the purpose of this systematic review is to synthesise evidence on the nature, impact, and socio-cultural drivers of epilepsy-related stigma in Nigeria. This review attempts to provide insights that could be useful for informing interventions to empower PLWE, reduce their burdens, improve their outcomes, and foster their inclusion in the Nigerian society.

**Method:**

Literature search was conducted using six electronic databases – APA PsycINFO, Google Scholar, JSTOR, MEDLINE, PubMed, and Scopus – to identify relevant studies published between 2011 and 2024. Qualitative and quantitative studies were included. A total of 10 studies met the inclusion criteria and were analysed using thematic and narrative syntheses.

**Results:**

The findings revealed that stigma is a multi-dimensional issue encompassing perceived, enacted, and internalised forms. Cultural misconceptions such as beliefs associating epilepsy with witchcraft, curses, and contagion were found to be the primary drivers of stigma. Economic barriers, gender-specific vulnerabilities, and social exclusion further perpetuate discrimination and healthcare disparities. Stigma is associated with social determinants of health, such as education, employment, and gender, to limit opportunities and quality of life for PLWE.

**Conclusion:**

This study highlights that stigma adversely affects PLWE, perpetuating marginalisation, social isolation, and healthcare inequalities.

**Contribution:**

Urgent action is required to implement culturally sensitive interventions, enhance healthcare policies, and increase awareness to address stigmatisation, ensuring equitable treatment and access.

## Introduction

Epilepsy is a neurological disorder characterised by recurring, unprovoked seizures which affects approximately 50 million people globally, with almost 80% residing in low- and middle-income countries (World Health Organization 2024). Active epilepsy is estimated to affect between 4 and 10 individuals per 1000 people in the general global population at any point in time (World Health Organization 2024). The estimated prevalence of active epilepsy is 9 per 1000 people, while lifetime prevalence is 16 per 1000 people in sub-Saharan Africa (Owolabi et al. 2020). Nigeria, with an estimated prevalence of 8 per 1000, bears 50% of sub-Saharan Africa’s epilepsy burden, making it a critical public health issue (Owolabi et al. 2020). Despite advancements in treatment offering options for seizure control (Perucca 2005), stigma remains a persistent challenge for people living with epilepsy (PLWE).

The stigma surrounding epilepsy manifests as enacted stigma (discrimination and prejudice), anticipated stigma (expectation of discrimination), and internalised stigma (self-stigmatisation) (Earnshaw & Chaudoir 2009; Scambler & Hopkins 1986). This stigma significantly affects the social, educational, and employment opportunities of PLWE, thereby reducing quality of life (Jacoby & Austin 2007). In a religio-culturally diverse country like Nigeria, with misconceptions often rooted in cultural and religious beliefs, PLWE suffer a double burden of living with the symptoms and combating social isolation. Families of PLWE may also face discrimination, while stigma deters many PLWE from seeking medical treatment and support services (Kariuki, Thomas & Newton 2021).

Despite growing global research, extensive evidence examining the experiences and socio-cultural factors contributing to epilepsy-related stigma in Nigeria remains significantly lacking, thereby limiting effective interventions. Culturally sensitive approaches are crucial in addressing stigma and improving the quality of life of PLWE in Nigeria’s ethnically diverse context (Oluwadele, Adediran & Sunkanmi 2023). This review seeks to bridge the knowledge gap on epilepsy-related stigma in Nigeria by investigating its nature, impact, and socio-cultural drivers. Insights from this review are intended to inform stigma-reduction policies and support the development of culturally sensitive interventions, promoting acceptance and support for PLWE in Nigeria.

## Methods

This study adopts critical theory to deconstruct societal norms perpetuating marginalisation and inequality, particularly for vulnerable groups like PLWE (Khaldi 2017). The focus of critical theory on social justice and emancipation enhances analysis of stigma rooted in cultural, historical, and institutional contexts. The adaptability of critical theory supports its use in mixed-method designs, integrating subjective and empirical approaches to relate personal experiences with societal patterns (Asghar 2013). This enables deeper examination to expose systemic issues, challenge the status quo, and promote transformative change, making it well-suited for addressing epilepsy-related stigma in Nigeria.

**FIGURE 1:**
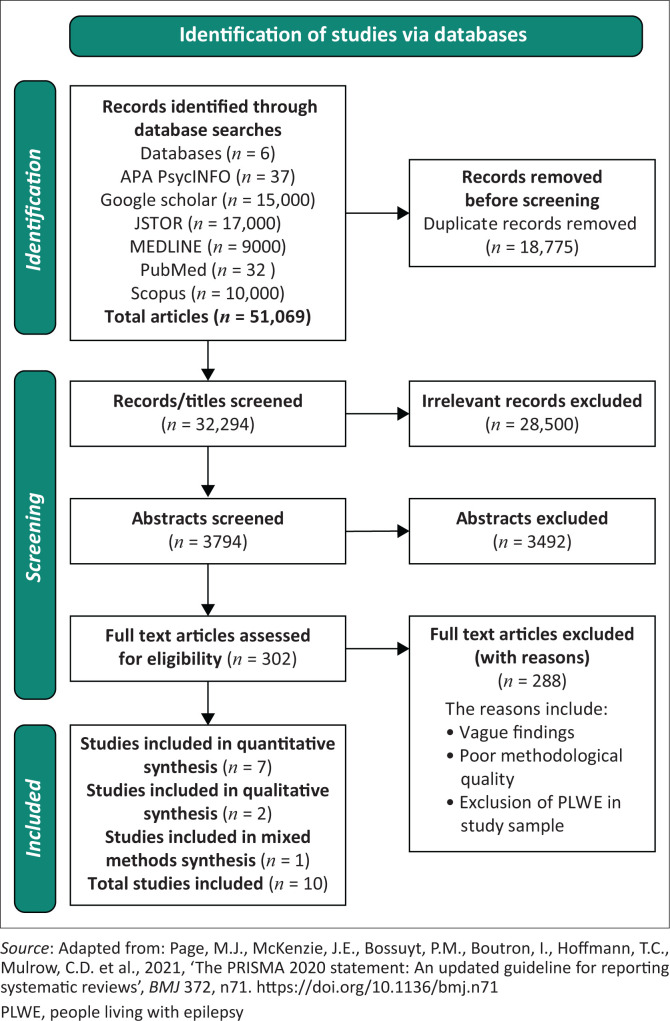
Preferred Reporting Items for Systematic Reviews and Meta-Analyses flowchart showing the study selection process.

The study systematically reviews evidence on the stigmatisation of PLWE in Nigeria, adopting both qualitative and quantitative methods for a comprehensive understanding of stigma associated with epilepsy. This approach combines broad statistical patterns with detailed narratives, leveraging their complementary strengths (Creswell 1999). The process follows the Preferred Reporting Items for Systematic Reviews and Meta-Analyses (PRISMA) guidelines, with study selection outlined in the accompanying flowchart (see Figure 1).

### Search strategy

Literature search was conducted using APA PsycINFO, Google Scholar, JSTOR, MEDLINE, PubMed, and Scopus to identify peer-reviewed studies published between 2011 and 2024. Keywords and Boolean operators included: ‘epilepsy’ AND ‘stigma’ OR ‘discrimination’ OR ‘stigmatisation’ AND ‘people’ OR ‘individuals’ AND ‘Nigeria’. Reference lists of identified studies were scanned for relevant titles, prompting additional searches.

#### Inclusion criteria

Inclusion criteria for the study are:

- Studies conducted on PLWE in Nigeria.
- Research addressing or evaluating stigma.
- Peer-reviewed English-language studies published between 2011 and 2024.

#### Exclusion criteria

Exclusion criteria for the study are:

- Studies conducted on PLWE outside Nigeria.
- Studies on epilepsy stigma not in English language.
- Studies on epilepsy stigma done beyond 2011.
- Studies focused on the medical treatment of epilepsy and review studies.

### Quality assessment

To ascertain the quality and methodological rigour of included studies, the qualitative and quantitative studies were assessed with the Critical Appraisal Skills Programme (CASP) tools appropriate for individual study designs (CASP 2024a, 2024b). Though this tool has been criticised for its lack of an objective scoring system and may depend on the skill of the reviewer (Masood et al. 2011), it still adequately assesses parameters needed for critical appraisal of studies, promotes critical thinking rather than a rigid scoring system and can flexibly be applied to a variety of study designs (Nadelson & Nadelson 2014). Also, the Mixed Methods Appraisal Tool (MMAT) was used to assess the mixed-methods study. Its reliability may be questioned as some sentences on the tool may be understood differently by different reviewers (Souto et al. 2015); however, it is still the only tool that does efficient critical appraisal of mixed-method study designs (Hong et al. 2018; Souto et al. 2015). Conflicts of interest were resolved by discussion and studies were excluded because of vague findings and poor quality.

### Data extraction

A Microsoft Excel spreadsheet was used to compile crucial information such as author(s), year, sample size, study design, demographics, and key findings.

### Data analysis

Thematic and narrative syntheses were used to analyse the qualitative and quantitative data. Thematic synthesis helped to identify common themes across studies, while narrative synthesis was used to highlight and compare study findings (Lucas et al. 2007).

### Ethical considerations

Ethical clearance to conduct this study was obtained from the Chair of the School of Allied & Public Health Research Ethics Sub-Committee, Faculty of Health, Medicine and Society at the University of Chester. The ethical clearance number is SAPH2425_057. Informed consent was not required as the primary data were not collected. Also, ethical standards were maintained by documenting the search strategy, citing sources, and accurately extracting and reporting findings.

## Review findings

Across all the included studies, the findings demonstrated that epilepsy-related stigma manifested through enacted stigma (direct discrimination such as avoidance, rejection, and hostile treatment), anticipated stigma (fear or expectation of being discriminated against, leading to secrecy and reduced participation), and internalised stigma (self-blame, shame, and diminished self-worth).

Ten studies were selected for qualitative and quantitative analysis of epilepsy-related stigma in Nigeria. The synthesis of findings revealed the following key themes.

### Synthesis of qualitative and quantitative findings

#### Cultural beliefs and misconceptions as drivers of epilepsy-related stigma

Epilepsy-related stigma stems from cultural misconceptions and limited awareness. Many respondents in the study by Adum et al. (2016) reported being labelled as ‘outcasts’ or ‘possessed’, resulting in social isolation. This labelling is a form of enacted stigma and beliefs about epilepsy being contagious usually led to avoidance of physical contact with PLWE. Arazeem, Adedolapo and Alabi (2022) noted that epilepsy is perceived as contagious, incurable or linked to supernatural causes such as ‘Jinn possession’ or ancestral curses called ‘*Ogun Idile*’ in Yoruba language spoken in South-West Nigeria. Jinn possession is an interesting concept rooted in Islamic religion which suggests that supernatural beings or ‘genies’ in a parallel realm come to possess the bodies of human beings and cause manifestations (Khalifa & Hardie 2005). These misconceptions normalise dehumanising stereotypes.

Komolafe et al. (2011) noted that women used ‘*giri*’ [spasms], ‘*warapa*’ [chronic wriggling], and ‘*ogun-oru*’ [nocturnal demon-related attacks] to describe epilepsy. These terms reinforced stigma, particularly among women with focal or nocturnal seizures who avoided using ‘*warapa*’ because of its association with incurability and societal rejection. Urban study participants attributed epilepsy to psychological causes such as chronic worrying and physical causes such as lack of immunisation, birth-related injuries, and head injuries. However, rural participants largely viewed it as spiritual, reflecting cultural and geographical divides.

Additionally, Komolafe et al. (2011) discovered that cultural beliefs linking epilepsy to curses and spiritual attacks perpetuate stigma, leading to broken relationships, spousal abandonment, and economic hardship for women. Study participants revealed that epilepsy often led to rejection by in-laws, forcing many women to return to their parental homes. In these situations, they faced compounded vulnerabilities, including abuse and exploitation, particularly when they were financially dependent and had limited social support.

Quantitative findings supported this pattern. Asindi and Eyong (2012) demonstrated that public knowledge about epilepsy is deeply rooted in false beliefs, which then promotes discrimination. About 40% of respondents were believed to be demon-possessed, 16% were called mad, and 4% were labelled as cursed. Achor et al. (2017) reported similar findings, with 43% called ‘possessed’ and 33% ‘witches’, promoting avoidance behaviours. Additionally, Ezeala-Adikaibe et al. (2018) highlighted that spiritual explanations of epilepsy reinforced stigma, negatively impacting the self-perception and social interactions of PLWE.

#### Social exclusion and disruption of quality of life as a result of epilepsy-related stigma

The term ‘public seizures’ was adopted to exemplify seizures occurring in public places as conceptualised in primary evidence. This was done to maintain fidelity with what was used in the primary evidence.

The stigma surrounding epilepsy profoundly affects various dimensions of the lives of PLWE, restricting their access to education, employment, healthcare, and social interactions. Arazeem et al. (2022) found that stigma and health challenges in educational settings caused delays, learning difficulties, and poor performance. According to the study, these delays occur because special needs education is not widely prioritised in schools, partly because of misunderstandings and negative attitudes towards the condition. Consequently, learning environments are rarely adapted to meet the individual needs of PLWE, leaving them without adequate educational support and making it more difficult to keep up academically with their peers. Also, the study revealed the presence of discrimination in workplaces. One participant revealed that colleagues attempted to use his condition as grounds for dismissal, falsely claiming that it threatened their well-being. Social exclusion is common, particularly during public seizures, where participants recounted being ignored because onlookers feared contagion (Arazeem et al. 2022).

Many participants in the study by Adum et al. (2016) reported experiencing humiliation, especially because of public seizures, leading to low self-esteem and secrecy. Self-blame and feelings of shame indicate internalised stigma. This led many PLWE to conceal their condition from people outside their immediate families to avoid rejection or embarrassment, demonstrating anticipation of stigma and thereby perpetuating social isolation.

Komolafe et al. (2011) found that paternal relatives often distanced themselves from women living with epilepsy, branding them as ‘witches’ or illegitimate, while families concealed the condition to avoid public shame, excluding them from social and cultural events. Economic repercussions were also highlighted in this study and included school dropouts, limited job prospects, and financial instability, particularly in food-related industries, where disclosure of epilepsy deterred customers.

Quantitative data reinforced these experiences. Achor et al. (2017) emphasised that stigmatisation excludes PLWE from full socio-economic participation, with 34.4% of respondents being avoided, 9.7% treated as inferior, and 16.1% felt they discomforted others. Family mistreatment affected 13%, while 3.2% faced discrimination from healthcare providers. Asindi and Eyong (2012) noted that 60% were mocked by peers, 50% considered school withdrawal because of embarrassment, and 56% were avoided during seizures.

Ezeala-Adikaibe et al. (2018) found a 23.1% unemployment rate among PLWE, linking limited opportunities to stigma and poverty. Participants faced social rejection, often driven by fear of contagion, alongside hostility from family and healthcare professionals. Onwuakagba et al. (2020) suggested that regardless of contributing factors, social rejection left PLWE feeling embarrassed and isolated, often lowering self-esteem and limiting community support. In line with this, Obiako et al. (2014) reported 28% experiencing discrimination and 5% indifference, with these attitudes impairing social interactions and increasing isolation.

Across studies, social exclusion functioned primarily as enacted stigma, while the associated emotional consequences manifested as internalised stigma.

#### Epilepsy-related stigma as a barrier to health-seeking behaviour

Stigmatisation negatively affects healthcare access for PLWE, with high costs and limited resources increasing reliance on traditional methods. Arazeem et al. (2022) noted that myths about epilepsy being contagious and incurable cause fear and avoidance behaviours, even among healthcare professionals, contributing to hostile attitudes in medical facilities. Some participants reported how rudely they were treated by health professionals which they linked to their illness. Fear of judgment also delayed healthcare-seeking, among study participants, pushing some towards traditional or spiritual healers. Furthermore, stigma disrupted personal relationships, resulting in marital rejection and divorce. Thus, stigma manifested in all three forms: enacted (hostile interactions), anticipated (fear of seeking medical care), and internalised (self-perceptions shaped by supernatural explanations).

Komolafe et al. (2011) highlighted that the belief in supernatural causes of epilepsy often leads women to seek help from traditional healers and spiritualists before turning to Western medical care. These treatments drained family resources, limiting access to anti-seizure medications. Stigma’s influence on healthcare is varied, involving hostile attitudes, financial barriers, fear, social isolation, lack of awareness, and gender-specific challenges as noted by Komolafe et al. (2011).

Quantitative findings supported these patterns: studies documented high levels of delayed treatment, underutilisation of biomedical services, and beliefs that hinder adherence to medical management. Together, these findings show that stigma, across all three forms, is a major determinant of healthcare access and treatment continuity in Nigeria.

### Socio-demographic factors associated with stigma

#### Gender

Achor et al. (2017) reported that 52.5% of females and 41.5% of males with epilepsy experienced perceived stigma, while 75% of females experienced enacted stigma compared to 62.3% of males. Similarly, Ezeala-Adikaibe et al. (2018) observed that severe stigma affected 24.3% of females versus 12.9% of males. Notably, males (62.9%) experienced milder forms of stigmatisation than females (51.4%). On the contrary, Oderinde and Ogunniyi (2020) found that severe internalised stigma was more prevalent among males (58.3%) than females (41.7%).

#### Level of education

Fawale et al. (2015) examined stigma among 99 adults in Ibadan, South-West Nigeria and found it to be more prevalent in those with secondary or lower education levels. Furthermore, Onwuakagba et al. (2020) showed that rural dwellers with minimal education (any level of education below secondary education) experienced higher stigma and poorer community integration. In their sample, 79.7% had secondary education, 8.6% had no formal education, and 5.7% had tertiary education. Achor et al. (2017) also linked primary or no education to heightened stigma, with 67.7% of such participants affected.

#### Employment status

Ezeala-Adikaibe et al. (2018) established a connection between stigmatising experiences and the inability to get or remain employed. Furthermore, Onwuakagba et al. (2020) found that participants who were apprentices or unemployed faced the most severe stigma and challenges with community reintegration. Obiako et al. (2014) discovered that 78% of 242 participants struggled to secure or retain jobs because of seizure-related absences, contributing to financial instability.

#### Relationship status

In the study by Oderinde and Ogunniyi (2020), single participants demonstrated higher levels of severe internalised stigma (56.5%) compared to married counterparts (43.5%), suggesting that partner or spousal support may lessen internalised stigma and provide protective advantages. Onwuakagba et al. (2020) observed that single and divorced participants reported higher levels of perceived stigma and lower levels of self-esteem and community integration than married ones. Achor et al. (2017) revealed that single, separated, divorced, and widowed individuals (48.7%) were more vulnerable to perceived stigma than married participants (35.3%).

### Seizure-related factors associated with stigma

#### Early onset

Fawale et al. (2015) associated a lower age of onset with higher perceived stigma. The findings by Onwuakagba et al. (2020) showed that individuals who developed epilepsy later in life experienced lower perceived stigma than those with earlier onset. Also, Ezeala-Adikaibe et al. (2018) identified that younger participants, particularly those under 10 years old, reported more severe stigma (95.6%) compared to those whose epilepsy began in adulthood (83.7%).

#### Visibility of epilepsy-related injuries

Achor et al. (2017) observed that 65.6% of participants with severe epilepsy-related injuries were more likely to report perceived stigma than those without such injuries. This finding was supported by Ezeala-Adikaibe et al. (2018) who pointed out a positive correlation between physical epilepsy-related injuries and enacted stigma, drawing a connection between the visibility of physical injuries and outward signs of seizures as a contributor to stigmatisation.

#### Public seizures and public knowledge of illness

In the study by Asindi and Eyong (2012) on the stigmatisation of children in South-South, Nigeria, 56% of the respondents reported being avoided during seizures, and almost 30% stated that teachers did not help probably because of fear of contagion. Ezeala-Adikaibe et al. (2018) further linked public seizure occurrences to enacted and perceived stigma. Achor et al. (2017) found that disclosure of epilepsy to non-family members heightened stigma, with 77.3% of such participants affected. On the other hand, 36.6% were stigmatised despite only disclosing to close family members.

## Implications and recommendations

Epilepsy-related stigma in Nigeria remains a pervasive public health issue, deeply rooted in cultural misconceptions, limited public awareness, and complex power dynamics (Hatzenbuehler 2016). Stigma perpetuated by systemic structures of power is shaped by cultural and societal factors and extends beyond mere ignorance (Campbell & Deacon 2006).

Misconceptions about epilepsy, including beliefs in its supernatural origins or contagion, reinforce stigma and exclusion, marginalise PLWE, and limit their access to education, employment, and healthcare (Adum et al. 2016; Arazeem et al. 2022). Nigeria’s underfunded and underdeveloped healthcare system, alongside healthcare professionals’ biases influenced by societal perceptions, compounds challenges for PLWE by limiting access to proper diagnosis, treatment, and care (Abah 2023; Owolabi et al. 2019). Furthermore, the interaction between prevalence and the different forms of epilepsy-related stigma is critical because the latter contribute to under-reporting, concealment, and delayed diagnosis, which may in turn underestimate the true burden of epilepsy (Oderinde & Ogunniyi 2020).

Our findings highlight the significant impact of social determinants of health (SDOH) on stigma-related experiences, with increased vulnerability among women, individuals with early-onset epilepsy, less educated individuals, and those experiencing frequent public seizures (Achor et al. 2017; Ezeala-Adikaibe et al. 2018; Fawale et al. 2015). While some studies link higher stigma in women to societal expectations and low socio-economic status (Achor et al. 2017; Ezeala-Adikaibe et al. 2018; Komolafe et al. 2011), Oderinde and Ogunniyi (2020) found that men experience more severe stigma, possibly because of perceived impact on their traditional roles as breadwinners. These findings show that epilepsy-related stigma may be context-based and shaped by societal gender norms.

Both qualitative and quantitative studies associate low education and unemployment with stigmatisation, consistent with prior findings (Adewumi, Oladipo & Adewuya 2020). Frequent seizures impair memory, productivity, and academics, worsened by teacher avoidance (Arazeem et al. 2022; Asindi & Eyong 2012). This reduces employment prospects, deepening financial strain, particularly when there is high national unemployment (Ezeala-Adikaibe et al. 2018).

Relationship status strongly correlates with epilepsy-related stigma, with unmarried PLWE experiencing heightened stigmatisation (Oderinde & Ogunniyi 2020; Onwuakagba et al. 2020). This is because of a lack of support systems, fear of isolation, and contributes to non-disclosure of the condition and high divorce rates (Komolafe et al. 2011). Furthermore, epilepsy-related injuries, public seizures, and disclosure worsen stigma by driving misconceptions about epilepsy as a dangerous and uncontrollable condition (Jacoby & Austin 2007). These often lead to social exclusion, negatively affecting healthcare access and quality of life. Furthermore, the unprofessional attitudes of health workers towards PLWE by Arazeem et al. (2022) is concerning as these professionals to an extent shape how epilepsy is perceived in the society and this can worsen stigma and drive PLWE to use unorthodox treatment which may have little impact on their improvement.

Overall, this review aimed to provide compelling evidence to drive effective reforms in epilepsy management in Nigeria. However, the scarcity of qualitative research and a higher number of hospital-based studies limited the in-depth exploration of the lived experiences of PLWE. Also, a meta-analysis could not be conducted because of the varied analytical focus of findings, thereby limiting the ability to draw stronger quantitative conclusions.

Public health interventions should extend beyond education to challenge the structural and systemic inequalities that marginalise PLWE, particularly vulnerable groups (Brown et al. 2019). Key strategies include advocating for better PLWE representation (Munjal et al. 2020), improving access to high-quality healthcare (World Health Organization 2022), and establishing support groups to reduce isolation (Elafros et al. 2013). Interventions must consider the interplay between SDOH and stigma-related experiences to ensure comprehensive support (Szaflarski 2014). Future research should prioritise community-based studies to better understand and address stigma at the grassroots level (Oderinde & Ogunniyi 2020). Collaborative efforts from key stakeholders such as policymakers, community and religious leaders, and healthcare professionals are important for developing and implementing policies that protect the rights and well-being of PLWE (Correa & Gutierrez 2024).

## Conclusion

This systematic review illuminates the pervasive and debilitating nature of epilepsy-related stigma in Nigeria, rooted in cultural misconceptions and amplified by socio-economic disparities. The findings reveal how stigma manifests as social exclusion, barriers to healthcare, and reduced quality of life, disproportionately affecting vulnerable groups such as women, the less educated, and those with early-onset epilepsy. Addressing this requires multi-faceted, culturally attuned strategies that go beyond individual education to dismantle systemic inequalities.

A coalition of 15 African countries, including Cameroon, Ethiopia, Eswatini, Kenya, Lesotho, Malawi, Mauritius, Sierra Leone, South Africa, South Sudan, Tanzania, The Gambia, Uganda, Zambia, and Zimbabwe, developed a toolkit that epilepsy advocates and stigma interventionists can adopt to ensure the human rights of PLWE are upheld across all social institutions (International Bureau for Epilepsy 2022). The toolkit can be contextualised to guide and support policy recommendations in Nigeria for: (1) implementing nationwide awareness campaigns to debunk myths, integrated into school curricula and media; (2) enhancing healthcare access through subsidised anti-seizure medications and training for bias-free care; (3) enacting anti-discrimination laws to protect PLWE in employment and education; (4) establishing community support groups to combat isolation; and (5) fostering partnerships with religious and traditional leaders to promote acceptance.

Incorporating the Health Stigma and Discrimination Framework (Stangl et al. 2019) as a behaviour change communication tool offers a structured pathway: targeting drivers like misinformation through mass media campaigns, mitigating manifestations via peer support networks, and improving outcomes by advocating for non-discriminatory policies. In Nigeria’s context, this could involve localised programmes, such as school-based education and collaborations with traditional healers, to shift attitudes and behaviours, ultimately dismantling barriers and fostering a society where PLWE thrive without marginalisation. Similarly, the Information-Motivation-Behavioural Skills (IMB) model offers a verifiable behavioural change communication framework applicable to health education on epilepsy management and stigma reduction (Xu & Wang 2023). This model posits that behaviour change occurs through providing accurate information (e.g. epilepsy as a medical condition), enhancing motivation (e.g. personal and social benefits of acceptance), and building skills (e.g. communication to challenge stigma). In Nigeria, IMB could guide interventions like workshops for communities, empowering participants to adopt supportive behaviours and reduce discrimination. By applying IMB, Nigeria can advance towards an inclusive society where PLWE enjoy equitable opportunities (Beyene et al. 2025).
